# Supplementary material for: Association between dyslipidemia and the risk of incident chronic kidney disease affected by genetic susceptibility: Polygenic risk score analysis
Source: PLoS One. 2024 Apr 16;19(4):e0299605. doi: 10.1371/journal.pone.0299605 (PMC11020804; doi:10.1371/journal.pone.0299605)

S1 Fig. Distribution of each lipid levels represented as quartiles.

Total cholesterol (mg/dL)

Min	25%	50%	75%	Max
23.24053	191.106	219.5669	249.3039	530.5104

LDL-C (mg/dL)

Min	25%	50%	75%	Max
10.28616	114.6945	136.6589	159.7835	378.8476

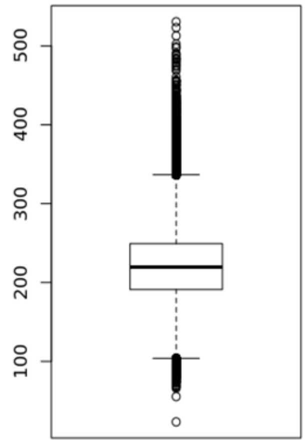

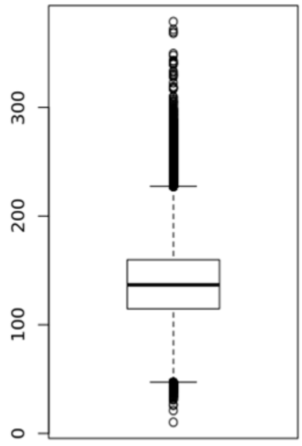

HDL-C (mg/dL)

Min	25%	50%	75%	Max
8.468677	45.55298	54.36968	65.08121	170.1856

TG (mg/dL)

Min	25%	50%	75%	Max
20.46058	92.64836	131.178	189.7254	998.9371

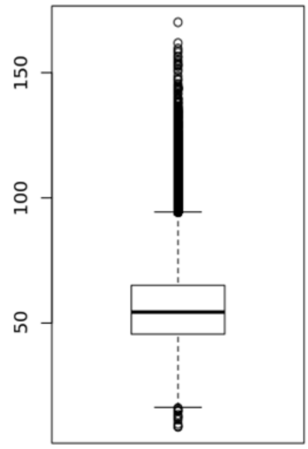

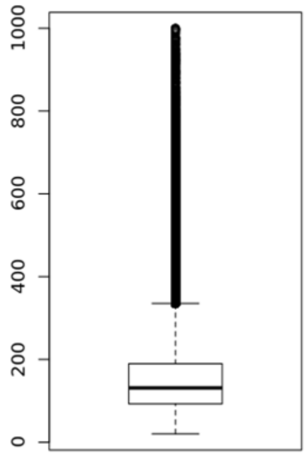

Supplement: S1 Fig — (PDF) [file pone.0299605.s001.pdf]
