# Supplementary material for: Association between dyslipidemia and the risk of incident chronic kidney disease affected by genetic susceptibility: Polygenic risk score analysis
Source: PLoS One. 2024 Apr 16;19(4):e0299605. doi: 10.1371/journal.pone.0299605 (PMC11020804; doi:10.1371/journal.pone.0299605)

**S2 Fig.** Restricted cubic spline curves for each lipid level. Hazard ratios are estimated using reference values for optimal levels in the 2015 Korean Guidelines for the Management of Dyslipidemia, which were 200 mg/dL mg/dL total cholesterol, 100 mg/dL LDL-C, 60 mg/dL HDL-C, and 150 mg/dL triglycerides

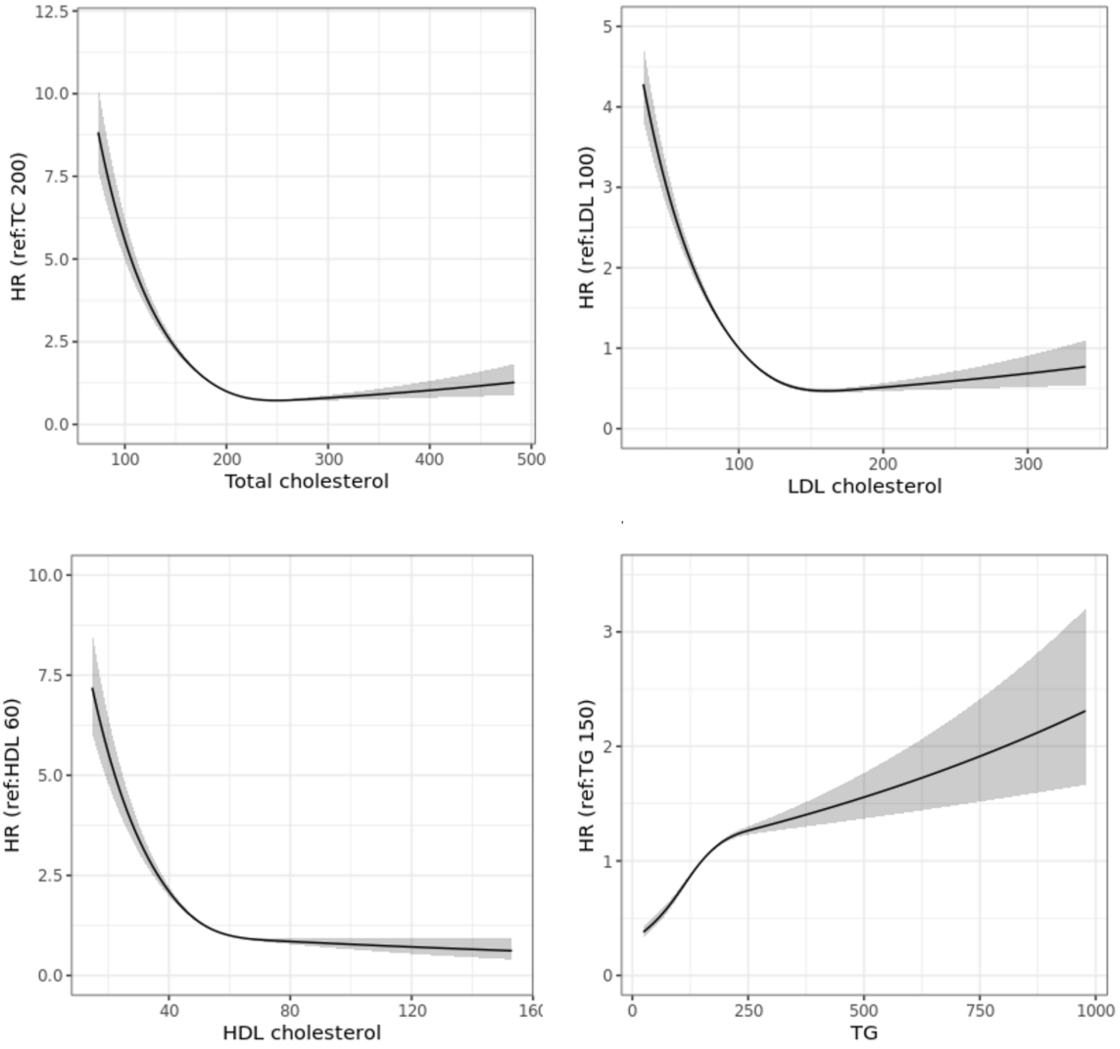

Supplement: S2 Fig — Hazard ratios are estimated using reference values for optimal levels in the 2015 Korean Guidelines for the Management of Dyslipidemia, which were 200 mg/dL mg/dL total cholesterol, 100 mg/dL LDL-C, 60 mg/dL HDL-C, and 150 mg/dL triglycerides. (PDF) [file pone.0299605.s002.pdf]
