# Supplementary material for: Association between dyslipidemia and the risk of incident chronic kidney disease affected by genetic susceptibility: Polygenic risk score analysis
Source: PLoS One. 2024 Apr 16;19(4):e0299605. doi: 10.1371/journal.pone.0299605 (PMC11020804; doi:10.1371/journal.pone.0299605)
Supplement: S1 Table — (PDF) [file pone.0299605.s003.pdf]

S1 Table. Comparison of performance for each PRS algorithm

PRS Algorithm	R	p-value	AIC
**P+T**	0.036	6.66E-15	25332.96
**C+T**	0.041	2.64E-18	25317.55
**LDpred2_inf**	0.051	5.59E-30	25263.7
**LDpred2_grid_0.03**	**0.061**	**1.18E-43**	**25199.84**
**LDpred2_grid_0.01**	**0.061**	3.43E-43	25201.92
**LDpred2_grid_0.3**	0.053	2.42E-32	25252.75
**LDpred2_grid_0.1**	0.057	1.10E-37	25227.91
**LDpred2_grid_1**	0.051	6.08E-30	25263.87
**LDpred2_auto**	0.059	3.72E-41	25211.6
**LASSOsum**	0.049	3.91E-26	25281.46
**PRScs**	0.051	1.72E-30	25261.3

P+T, pruning and thresholding; C+T, clumping and thresholding; LDpred2_inf, LDpred2 infinitesimal model; LDpred2_grid, LDpred2 grid model (The appended numbers signify the proportions of causal variants); LDpred2_auto, LDpred2 auto model
