# Supplementary material for: Association between dyslipidemia and the risk of incident chronic kidney disease affected by genetic susceptibility: Polygenic risk score analysis
Source: PLoS One. 2024 Apr 16;19(4):e0299605. doi: 10.1371/journal.pone.0299605 (PMC11020804; doi:10.1371/journal.pone.0299605)
Supplement: S2 Table — (PDF) [file pone.0299605.s004.pdf]

S2 Table. Univariable analysis using Cox regression model for incident CKD

Variable	HR (95% CI)	P-value
Age	1.119 (1.114-1.125)	**<0.001**
Sex		
Female	Ref.	
Male	1.288 (1.214-1.366)	**<0.001**
Body mass index	1.086 (1.081-1.091)	**<0.001**
Comorbidity (Yes)		
Diabetes	5.291 (4.915-5.695)	**<0.001**
Hypertension	2.452 (2.310-2.603)	**<0.001**
Dyslipidemia	4.048 (3.815-4.295)	**<0.001**
Alcohol intake frequency		
Daily or almost daily	Ref.	
Three or four times a week	0.856 (0.776-0.944)	**0.002**
Once or twice a week	1.069 (0.976-1.171)	0.150
One to three times a month	1.188 (1.063-1.328)	**0.002**
Special occasions only	1.807 (1.635-1.997)	**<0.001**
Never	2.108 (1.889-2.352)	**<0.001**
Smoking status		
Never	Ref.	
Previous	1.542 (1.448-1.642)	**<0.001**
Current	1.410 (1.281-1.553)	**<0.001**
Lipid levels		
Total cholesterol	0.662 (0.642-0.683)	**<0.001**
LDL-C	0.685 (0.664-0.707)	**<0.001**
HDL-C	0.664 (0.641-0.689)	**<0.001**
Triglyceride	1.238 (1.210-1.267)	**<0.001**
Polygenic Risk Score (continuous)	1.230 (1.195-1.267)	**<0.001**
Polygenic Risk Score tertile		
Low	Ref.	
Intermediate	1.268 (1.173-1.370)	**<0.001**
High	1.626 (1.510-1.750)	**<0.001**

LDL-C, low density lipoprotein cholesterol; HDL-C, high density lipoprotein cholesterol; PRS, polygenic risk score; HR, hazard ratio; CI, confidence interval; SE, standard error; Ref., reference category.

Hazard ratios were reported as per 1-SD change for lipid levels.
