# Supplementary material for: Association between dyslipidemia and the risk of incident chronic kidney disease affected by genetic susceptibility: Polygenic risk score analysis
Source: PLoS One. 2024 Apr 16;19(4):e0299605. doi: 10.1371/journal.pone.0299605 (PMC11020804; doi:10.1371/journal.pone.0299605)
Supplement: S3 Table — (PDF) [file pone.0299605.s005.pdf]

S3 Table. Subgroup analysis for incident CKD stratified by statin use

	No statin use (N=318,435)		Statin use (N=55,088)	
	**HR (95% CI)**	**P-value**	**HR (95% CI)**	**P-value**
Total Cholesterol	0.884 (0.847-0.923)	**<0.001**	0.934 (0.875-0.997)	**0.042**
LDL-C	0.884 (0.847-0.922)	**<0.001**	0.936 (0.874-1.002)	0.056
HDL-C	0.881 (0.836-0.929)	**<0.001**	0.890 (0.830-0.955)	**0.001**
Triglyceride	1.078 (1.039-1.119)	**<0.001**	1.064 (1.019-1.111)	**0.005**

LDL-C, low density lipoprotein cholesterol; HDL-C, high density lipoprotein cholesterol; HR, hazard ratio; CI, confidence interval; SE, standard error.
Models including each lipid level (separate model for each lipid category). Adjusted for age, sex, BMI, alcohol intake frequency, smoking status, comorbidities (diabetes, hypertension), fibrate use, fasting time and eGFR.
Hazard ratios were reported as per 1-SD change for lipid levels.
