# Supplementary material for: Association between dyslipidemia and the risk of incident chronic kidney disease affected by genetic susceptibility: Polygenic risk score analysis
Source: PLoS One. 2024 Apr 16;19(4):e0299605. doi: 10.1371/journal.pone.0299605 (PMC11020804; doi:10.1371/journal.pone.0299605)
Supplement: S4 Table — (PDF) [file pone.0299605.s006.pdf]

S4 Table. Baseline characteristics of study population without history of dyslipidemia or statin use stratified by LDL-C and total cholesterol

**Variable**	**LDL-C < 100mg/dL (N=20,694)**	**LDL-C ≥ 100mg/dL (N=287,599)**	**P-value**	**Total cholesterol < 200mg/dL (N=75,710)**	**Total cholesterol ≥ 200mg/dL (N=233,101)**	**P-value**
Age (years)	51.60±8.26	55.88±7.91	**<0.001**	52.93±8.38	56.46±7.68	**<0.001**
Sex, n(%)			**<0.001**			**<0.001**
Female	12537 (60.58%)	163946 (57.01%)		39012 (51.53%)	137724 (59.08%)	
Male	8157 (39.42%)	123653 (42.99%)		36698 (48.47%)	95377 (40.92%)	
Body mass index	25.46±4.72	27.04±4.57	**<0.001**	26.53±4.88	27.06±4.49	**<0.001**
Comorbidities						
Diabetes	647 (3.13%)	3199 (1.11%)	**<0.001**	1831 (2.42%)	2023 (0.87%)	**<0.001**
Hypertension	3031 (14.65%)	54547 (18.97%)	**<0.001**	12862 (16.99%)	44800 (19.22%)	**<0.001**
Smoking status, n(%)			**<0.001**			**<0.001**
Never	11808 (57.22%)	161178 (56.22%)		43639 (57.81%)	129635 (55.79%)	
Previous	6486 (31.43%)	96172 (33.54%)		23784 (31.51%)	79054 (34.02%)	
Current	2343 (11.35%)	29350 (10.24%)		8066 (10.69%)	23677 (10.19%)	
Serum creatinine (mg/dL)	0.79±0.15	0.80±0.15	**<0.001**	0.81±0.15	0.79±0.15	**<0.001**
eGFR (ml/min/1.73m2)	95.50±12.23	92.24±11.67	**<0.001**	94.30±12.10	91.87±11.56	**<0.001**
C-reactive Protein (mg/L)	2.62±5.50	2.50±4.14	**0.001**	2.66±5.16	2.46±3.90	**<0.001**
Serum albumin (g/L)	44.52±2.71	45.29±2.57	**<0.001**	44.69±2.61	45.42±2.55	**<0.001**
Polygenic risk score	0.00±1.00	0.00±1.00	0.970	0.00±1.00	-0.01±1.00	**0.043**
Polygenic risk score tertile			0.946			0.162
Low	6933 (33.50%)	96258 (33.47%)		25184 (33.26%)	78171 (33.54%)	
Intermediate	6918 (33.43%)	95922 (33.35%)		25205 (33.29%)	77811 (33.38%)	

High	6843 (33.07%)	95419 (33.18%)		25321 (33.44%)	77119 (33.08%)	
Incident CKD			0.483			0.457
No	20544 (99.28%)	285380 (99.23%)		75144 (99.25%)	231293 (99.22%)	
Yes	150 (0.72%)	2219 (0.77%)		566 (0.75%)	1808 (0.78%)	

CKD, chronic kidney disease; eGFR, estimated glomerular filtration rate.

Data was reported as mean ± SD for continuous variables and n(%) for categorical variables. P-value was computed by t-test for continuous variables and chi-square test or Fisher's exact test for categorical variables as appropriate.
