# Supplementary material for: Association between dyslipidemia and the risk of incident chronic kidney disease affected by genetic susceptibility: Polygenic risk score analysis
Source: PLoS One. 2024 Apr 16;19(4):e0299605. doi: 10.1371/journal.pone.0299605 (PMC11020804; doi:10.1371/journal.pone.0299605)
Supplement: Attachment — Submitted filename: Rebuttal letter.docx [file pone.0299605.s007.docx]

**Comments from the Editor and Reviewers**

**Editor comments:**
Thank you for submitting your manuscript to PLOS ONE. After careful consideration, we feel that it has merit but does not fully meet PLOS ONE’s publication criteria as it currently stands. Therefore, we invite you to submit a revised version of the manuscript that addresses the points raised during the review process.

==============================
ACADEMIC EDITOR:

The manuscript is interesting but will require further reworking and a major revision.

While they recognize the potential interest of the subject studied, the reviewers raised a number of important issues that need to be properly addressed.

==============================

Please submit your revised manuscript by Nov 23 2023 11:59PM. If you will need more time than this to complete your revisions, please reply to this message or contact the journal office at [plosone@plos.org](mailto:plosone@plos.org). When you're ready to submit your revision, log on to <https://www.editorialmanager.com/pone/> and select the 'Submissions Needing Revision' folder to locate your manuscript file.

Please include the following items when submitting your revised manuscript:

- A rebuttal letter that responds to each point raised by the academic editor and reviewer(s). You should upload this letter as a separate file labeled 'Response to Reviewers'.
- A marked-up copy of your manuscript that highlights changes made to the original version. You should upload this as a separate file labeled 'Revised Manuscript with Track Changes'.
- An unmarked version of your revised paper without tracked changes. You should upload this as a separate file labeled 'Manuscript'.

If you would like to make changes to your financial disclosure, please include your updated statement in your cover letter. Guidelines for resubmitting your figure files are available below the reviewer comments at the end of this letter.

If applicable, we recommend that you deposit your laboratory protocols in [protocols.io](http://protocols.io/) to enhance the reproducibility of your results. Protocols.io assigns your protocol its own identifier (DOI) so that it can be cited independently in the future. For instructions see: <https://journals.plos.org/plosone/s/submission-guidelines#loc-laboratory-protocols>. Additionally, PLOS ONE offers an option for publishing peer-reviewed Lab Protocol articles, which describe protocols hosted on [protocols.io](http://protocols.io/). Read more information on sharing protocols at <https://plos.org/protocols?utm_medium=editorial-email&utm_source=authorletters&utm_campaign=protocols>.

**Reviewers' comments:**

**Reviewer #1:**

The manuscript titled "Association between dyslipidemia and the risk of incident chronic kidney disease affected by genetic susceptibility: polygenic risk score analysis" delves into the intricate relationship between dyslipidemia, genetic predisposition (quantified using a polygenic risk score, or PRS), and the onset of chronic kidney disease (CKD). Drawing data from the extensive UK Biobank cohort, the study meticulously constructs a PRS to encapsulate individual genetic susceptibility. Through multivariable Cox regression models, the authors unveiled that heightened triglyceride levels and diminished levels of total cholesterol, LDL-C, and HDL-C amplify the risk of CKD. Moreover, the PRS emerged as a potent predictor of CKD, with a notable interaction between triglyceride levels and PRS, especially in the low-PRS group, signifying a heightened CKD risk.

This research bridges genetic markers and phenotypic indicators to shed light on CKD risk. The methodological rigor, combined with the vastness of the dataset, instills confidence in the findings. However, while the core analysis is robust, the manuscript could benefit from a deeper exploration of underlying mechanisms and broader implications, ensuring the results resonate more profoundly within clinical and research landscapes.

1 - The introduction effectively provides a broad context of the topic at hand. However, the inconsistencies related to dyslipidemia's impact on renal function could be more clearly elucidated. Additionally, delving deeper into the clinical implications, particularly those concerning statin interventions, would enrich the discussion. To enhance the scientific rigor of the introduction, it's essential to emphasize the study's significance, highlight gaps in the existing literature, and clearly outline the foundational rationale behind the research.

**Response:** Thank you for the important comment. Following the valuable comments from the reviewer, we have added additional descriptions in the Introduction section to emphasize the significance of the present study based on inconsistencies in the existing literature as follows: “Dyslipidemia, despite its strong association with cardiovascular disease, diabetes mellitus, and hypertension, studies have shown inconsistent results regarding its impact on the onset and prognosis of CKD. In observational studies conducted on the general population, elevated levels of total cholesterol and LDL-cholesterol (LDL-C), as well as low levels of HDL-cholesterol (HDL-C), have exhibited varying associations with renal function decline, showing significant correlations in some studies and lacking such associations in others [1-3]. Moreover, randomized controlled trials comparing renal outcomes after the use of lipid-lowering agents such as statins, excluding patient groups with cardiovascular disease, have consistently shown that the use of these agents have no effects on the outcomes [4, 5]. Furthermore, observational studies of CKD populations have demonstrated that cholesterol and triglyceride levels are unrelated to the onset of end-stage kidney disease (ESKD), except in the low albuminuria subgroup in one study. [6-8]. Currently, lipid-lowering management is recommended for CKD populations primarily for the prevention of cardiovascular disease [9]. Considering the close relationship between CKD and cardiovascular disease, these inconsistencies make it necessary to evaluate each individual's risk of CKD from dyslipidemia for proper management.”

2 - Considering the comprehensive methodology you outlined for PRS construction, how did the LDpred2 algorithm's performance compare to the other four algorithms in terms of specificity, sensitivity, and overall accuracy? Could you provide a table or figure that visually contrasts the performance metrics of each algorithm, and elaborate on the primary factors that led to the selection of LDpred2 as the optimal PRS model over the others?

**Response:** Thank you for the valuable comment. Following the comment, we present a comparison of each PRS algorithm for selecting optimal PRS as a supplemental table. We utilized five different algorithms: P+T, C+T, LDpred2, LASSOsum, and PRScs, to calculate the PRS. Specifically, for LDpred2 PRS, we employed a grid model (LDpred2_grid) with proportions of causal variants set at 0.03, 0.01, 0.3, 0.1, and 1, and also utilized an infinitesimal model (LDpred2_inf) and an auto model (LDpred2_auto). Supplemental Table 1 (S1 table) visualizes the performance comparison for each PRS.

**S1 Table. Comparison of performance for each PRS algorithm**

| PRS Algorithm | R | p-value | AIC |
| --- | --- | --- | --- |
| P+T | 0.036 | 6.66E-15 | 25332.96 |
| C+T | 0.041 | 2.64E-18 | 25317.55 |
| LDpred2_inf | 0.051 | 5.59E-30 | 25263.7 |
| LDpred2_grid_0.03 | **0.061** | **1.18E-43** | **25199.84** |
| LDpred2_grid_0.01 | **0.061** | 3.43E-43 | 25201.92 |
| LDpred2_grid_0.3 | 0.053 | 2.42E-32 | 25252.75 |
| LDpred2_grid_0.1 | 0.057 | 1.10E-37 | 25227.91 |
| LDpred2_grid_1 | 0.051 | 6.08E-30 | 25263.87 |
| LDpred2_auto | 0.059 | 3.72E-41 | 25211.6 |
| LASSOsum | 0.049 | 3.91E-26 | 25281.46 |
| PRScs | 0.051 | 1.72E-30 | 25261.3 |

P+T, pruning and thresholding; C+T, clumping and thresholding; LDpred2_inf, LDpred2 infinitesimal model; LDpred2_grid, LDpred2 grid model (The appended numbers signify the proportions of causal variants); LDpred2_auto, LDpred2 auto model

After careful evaluation, we have selected LDpred2_grid_0.03 as the optimal PRS, which demonstrated the highest correlation (R=0.061), significance (p=1.18E-43), and the lowest Akaike information criterion (AIC = 25199.84). In the Method section, we added these descriptions, “In the PRS tuning process, we finally chose a model using LDpred2 as an optimal PRS based on the highest correlation (R), significance (p-value), and the lowest Akaike information criterion (AIC) in regression. The comparison of each PRS algorithm is visualized in S1 Table.”

3 - How might the exclusions, particularly those concerning ethnicity and relatedness, affect the broader applicability of the study's findings to diverse populations? Considering the exclusions based on non-white ethnicity and relatedness up to the 3rd degree, have you considered conducting a Principal Component Analysis (PCA) to illustrate the ancestral background of both the included and excluded participants? Such an analysis could provide insights into the genetic diversity of the cohort and further contextualize the potential generalizability of your findings.

**Response:** Thank you for your valuable suggestion. The primary objective of our manuscript is to explore the impact of genetic factors on the relationship between dyslipidemia and chronic kidney disease (CKD) risk, using polygenic risk scores (PRS) in a European population. We utilized the UK Biobank data, which encompasses a large number of subjects, and therefore chose to focus exclusively on the European population to enhance the robustness of our analyses. Additionally, in light of your comment, we reviewed the multidimensional scaling (MDS) plot and concluded that focusing on the European population is indeed the most suitable approach for our study.

4 - Your study unveils the potential of PRS in capturing the risk of incident kidney diseases and emphasizes its interaction with lipid levels, particularly triglycerides. Given this novel insight, are there plans to further investigate the specific genetic loci or pathways that might be mediating this interaction?

**Response:** Thank you for the insightful comment. The reviewer highlighted the necessity of investigating specific risk loci or pathways involved in the interaction between dyslipidemia and genetic risk for CKD. While the reviewer’s suggestion is indeed important, it is considered that an extensive analysis in this direction would require resources beyond the current scope of our research. However, recognizing the significance of this intriguing topic, further investigation is deemed necessary to elucidate and expand upon it. In the Discussion section, we added these descriptions, “Finally, in the present study, since the mechanism underlying the interaction between triglycerides and genetic factors is not elucidated, additional investigations are necessary to explore the specific genetic loci or pathways that mediate the interaction.”

5 - Would it be possible to produce a Manhattan plot that illustrates the risk alleles (SNPs) linked to incident CKD within the UK Biobank cohort?

**Response:** Thank you for your valuable comment. The primary objective of our research is to examine the interaction between genetic factors for CKD, manifested by PRS, and the influence of dyslipidemia on CKD. Manhattan plots for CKD risk alleles have already been presented in several GWAS utilizing various cohorts. Consequently, we acknowledge that providing additional Manhattan plots in our study may have a relatively lower impact in terms of relevance to the research topic and novelty.

We added the following descriptions in Introduction section, “Several GWAS have identified risk alleles associated with the development of CKD, visualized in Manhattan plots [15, 16].”, and cited two articles presenting Manhattan plots for CKD risk alleles as references.

15.	Lin BM, Nadkarni GN, Tao R, Graff M, Fornage M, Buyske S, et al. Genetics of Chronic Kidney Disease Stages Across Ancestries: The PAGE Study. Front Genet. 2019;10:494. Epub 2019/06/11. http://doi.org/10.3389/fgene.2019.00494

16.	Sugawara Y, Hirakawa Y, Nagasu H, Narita A, Katayama A, Wada J, et al. Genome-wide association study of the risk of chronic kidney disease and kidney-related traits in the Japanese population: J-Kidney-Biobank. J Hum Genet. 2023;68(2):55-64. Epub 2022/11/21. http://doi.org/10.1038/s10038-022-01094-1

6 - Given that the UK Biobank's population is predominantly white, how imperative is it to replicate this study across diverse ethnic groups to validate the universal applicability of your findings?

**Response:** Thank you for your important comment. The reviewer highlighted the necessity for validation regarding the universal applicability across diverse ethnic groups. The authors fully agree with these concerns. Consequently, we are currently undertaking a PRS study utilizing the East Asian population from the Korean Genome and Epidemiology Study (KoGES) and BioBank Japan (BBJ). In addition, we are trying to develop a multi-ethnicity CKD PRS by simultaneously utilizing GWAS summary statistics from Asian and Western populations. Unfortunately, we have not established yet the full datasets, so it is not practical to validate the results of this study in other ethnic groups in the limited timeframe available. We've added these limitations to the Discussion section as follows : “First, the study population in the UK Biobank was limited to white individuals who live in UK. Because genetic effects on incident CKD can vary according to ethnicity, it is necessary to confirm whether the effects of PRS, lipid profiles, and their interactions on CKD are the same in other populations or ethnicities.”

**Reviewer #2**:

This is an interesting study that evaluated the association between lipid levels, polygenic risk score and incident CKD in a large population. The authors found that high triglyceride was associated with incident CKD in the low-PRS group.
This association was significant after adjustment to diabetes and statin use, but the model was not adjusted to fibrate use. This is the main limitation of the study knowing that fibrate use for hypertrigyceridemia can induce an increase in serum creatinine. Could this confounding variable be added to the regression model?

**Response:** Thank you for the valuable comment. The authors acknowledge these concerns. We conducted a re-analysis by incorporating a variable of fibrate use into the multivariable model, and additionally included fasting time as additional covariate, considering the significant relationship between fasting status and triglyceride level. Subsequent re-analysis results revealed that the impact of each lipid level on CKD and the interactions between lipid levels and PRS were largely consistent with the initial analysis. Consequently, corresponding adjustments were made to Table 1, 2, 3, 4, and Figure 2 to reflect the updated content. The description of these findings in the Results section has also been revised.

**Table 1. Baseline characteristics of study population according to the incident CKD during follow-up period.**

| **Variable** | **Total** | **No incident CKD** | **Incident CKD** | **P-value** | **Missing data** |
| --- | --- | --- | --- | --- | --- |
|  | **(N=373,523)** | **(N=369,099)** | **(N=4,424)** |  | **N (%)** |
| Age (years) | 56.57±8.00 | 56.51±8.00 | 62.17±5.98 | **<0.001** | 0 (0%) |
| Sex, n(%) |  |  |  | **<0.001** | 0 (0%) |
| Female | 201731 (54.01%) | 199619 (54.08%) | 2112 (47.74%) |  |  |
| Male | 171792 (45.99%) | 169480 (45.92%) | 2312 (52.26%) |  |  |
| Body mass index | 27.34±4.74 | 27.31±4.72 | 29.74±5.61 | **<0.001** | 1176 (0.31%) |
| Alcohol intake frequency, n(%) |  |  |  | **<0.001** | 286 (0.08%) |
| Daily or almost daily | 80075 (21.45%) | 79259 (21.49%) | 816 (18.47%) |  |  |
| Three or four times a week | 89826 (24.07%) | 89042 (24.14%) | 784 (17.75%) |  |  |
| Once or twice a week | 97769 (26.19%) | 96704 (26.22%) | 1065 (24.11%) |  |  |
| One to three times a month | 41413 (11.10%) | 40912 (11.09%) | 501 (11.34%) |  |  |
| Special occasions only | 39570 (10.60%) | 38844 (10.53%) | 726 (16.44%) |  |  |
| Never | 24584 (6.59%) | 24059 (6.52%) | 525 (11.89%) |  |  |
| Smoking status, n(%) |  |  |  | **<0.001** | 1287 (0.34%) |
| Never | 201352 (54.09%) | 199423 (54.21%) | 1929 (43.86%) |  |  |
| Previous | 131742 (35.39%) | 129801 (35.29%) | 1941 (44.13%) |  |  |
| Current | 39142 (10.52%) | 38614 (10.50%) | 528 (12.01%) |  |  |
| Baseline comorbidities, n(%)† |  |  |  |  |  |
| Diabetes | 17200 (4.60%) | 16315 (4.42%) | 885 (20.00%) | **<0.001** | 0(0%) |
| Hypertension | 86187 (23.07%) | 84319 (22.84%) | 1868 (42.22%) | **<0.001** | 0(0%) |
| Dyslipidemia | 62871 (16.83%) | 60892 (16.50%) | 1979 (44.73%) | **<0.001** | 0(0%) |
| Medication, n(%)† |  |  |  |  |  |
| Diabetes | 9818 (2.63%) | 9222 (2.50%) | 596 (13.47%) | **<0.001** | 0(0%) |
| Hypertension | 56424 (15.11%) | 54459 (14.75%) | 1965 (44.42%) | **<0.001** | 0(0%) |
| Statin use, n(%) | 55088 (14.75%) | 53352 (14.45%) | 1736 (39.24%) | **<0.001** | 0 (0%) |
| Fibrate use, n(%) | 731 (0.20%) | 683 (0.19%) | 48 (1.08%) | **<0.001** | 0 (0%) |
| Serum creatinine (mg/dL) | 0.80±0.15 | 0.80±0.15 | 0.93±0.16 | **<0.001** | 0 (0%) |
| eGFR (ml/min/1.73m2) | 91.66±11.88 | 91.83±11.77 | 77.74±12.30 | **<0.001** | 0 (0%) |
| Lipid levels (mg/dL) |  |  |  |  |  |
| Total cholesterol | 221.24±43.99 | 221.45±43.88 | 204.20±49.14 | **<0.001** | 103 (0.03%) |
| LDL-C | 138.24±33.48 | 138.38±33.41 | 126.40±36.44 | **<0.001** | 715 (0.19%) |
| HDL-C | 56.32±14.81 | 56.38±14.80 | 51.12±14.40 | **<0.001** | 31643 (8.47%) |
| Triglyceride | 154.43±90.33 | 154.14± 90.13 | 179.16±102.94 | **<0.001** | 163 (0.04%) |
| Fasting time (hours) | 3.76±2.39 | 3.76±2.38 | 3.95±2.56 | **<0.001** | 80 (0.02%) |
| Polygenic risk score tertile |  |  |  | **<0.001** | 0 (0%) |
| Low | 124508 (33.33%) | 123370 (33.42%) | 1138 (25.72%) |  |  |
| Intermediate | 124507 (33.33%) | 123066 (33.34%) | 1441 (32.57%) |  |  |
| High | 124508 (33.33%) | 122663 (33.23%) | 1845 (41.70%) |  |  |

CKD, chronic kidney disease; eGFR, estimated glomerular filtration rate; LDL-C, low density lipoprotein cholesterol; HDL-C, high density lipoprotein cholesterol

Data was reported as mean ± SD for continuous variables and n(%) for categorical variables.
P-value was computed by t-test for continuous variables and chi-square test or Fisher's exact test for categorical variables as appropriate.
†Number of samples with each comorbidity or taking medication is give\

**Table 2. Multivariable analysis using Cox regression model for incident CKD.**

| **Variables** | **HR (95% CI)** | | **P-value** |
| --- | --- | --- | --- |
| **Polygenic risk score ^a^** |  |  |  |
| PRS (continuous variable) | 1.076 | (1.043-1.110) | **<0.001** |
| PRS tertile |  |  |  |
| Low |  | Ref. |  |
| Intermediate | 1.091 | (1.009-1.181) | **0.030** |
| High | 1.204 | (1.116-1.299) | **<0.001** |
| **Lipid levels ^b^** |  | |  |
| Total cholesterol | 0.896 | (0.865-0.929) | **<0.001** |
| LDL-C | 0.896 | (0.865-0.929) | **<0.001** |
| HDL-C | 0.876 | (0.840-0.913) | **<0.001** |
| Triglyceride | 1.082 | (1.052-1.113) | **<0.001** |

LDL-C, low density lipoprotein cholesterol; HDL-C, high density lipoprotein cholesterol; PRS, polygenic risk score; HR, hazard ratio; CI, confidence interval; SE, standard error; Ref., reference category.

Hazard ratios were reported as per 1-SD change for lipid levels.

a: Models including polygenic risk score (continuous variable or tertile). Adjusted for age, sex, BMI, alcohol intake frequency, smoking status, comorbidities (diabetes, hypertension), statin use, fibrate use, fasting time, baseline eGFR, and top 10 genetic principal components.
b: Models including each lipid level (separate model for each lipid category). Adjusted for age, sex, BMI, alcohol intake frequency, smoking status, comorbidities (diabetes, hypertension), statin use, fibrate use, fasting time and baseline eGFR

**Table 3. Multivariable analysis for interaction between lipid levels and PRS.**

| **Variables** | **HR (95% CI)** | | **P-value** | |
| --- | --- | --- | --- | --- |
| Total cholesterol | 0.877 | (0.827-0.931) | | **<0.001** |
| Intermediate PRS | 1.098 | (1.009-1.194) | | **0.029** |
| High PRS | 1.222 | (1.127-1.325) | | **<0.001** |
| Intermediate PRS * Total cholesterol | 1.015 | (0.944-1.092) | | 0.684 |
| High PRS * Total cholesterol | 1.039 | (0.969-1.114) | | 0.280 |
| LDL-C | 0.876 | (0.825-0.931) | | **<0.001** |
| Intermediate PRS | 1.095 | (1.007-1.190) | | **0.034** |
| High PRS | 1.224 | (1.129-1.326) | | **<0.001** |
| Intermediate PRS * LDL-C | 1.008 | (0.935-1.086) | | 0.840 |
| High PRS * LDL-C | 1.047 | (0.975-1.124) | | 0.205 |
| HDL-C | 0.820 | (0.762-0.881) | | **<0.001** |
| Intermediate PRS | 1.123 | (1.028-1.226) | | **0.010** |
| High PRS | 1.219 | (1.119-1.328) | | **<0.001** |
| Intermediate PRS * HDL-C | 1.123 | (1.026-1.228) | | **0.012** |
| High PRS * HDL-C | 1.073 | (0.985-1.170) | | 0.107 |
| Triglyceride | 1.152 | (1.094-1.212) | | **<0.001** |
| Intermediate PRS | 1.121 | (1.033-1.215) | | **0.006** |
| High PRS | 1.226 | (1.133-1.327) | | **<0.001** |
| Intermediate PRS * Triglyceride | 0.899 | (0.838-0.965) | | **0.003** |
| High PRS * Triglyceride | 0.934 | (0.874-0.997) | | **0.041** |

LDL-C, low density lipoprotein cholesterol; HDL-C, high density lipoprotein cholesterol; PRS, polygenic risk score; HR, hazard ratio; CI, confidence interval; SE, standard error.

Hazard ratios were reported as per 1-SD change for lipid levels.

Low PRS group was a reference category.
Models including each lipid level, PRS tertile, and multiplicative interaction term between them. Adjusted for age, sex, BMI, alcohol intake frequency, smoking status, comorbidities (diabetes, hypertension), statin use, fibrate use, fasting time, baseline eGFR, the top 10 genetic principal components.

**Table 4. Subgroup analysis using Cox regression model for incident CKD according to PRS tertiles.**

|  |  | **Low PRS (N=124,508)** | | |  | **Intermediate PRS (N=124,507)** | | |  | **High PRS (N=124,508)** | | |
| --- | --- | --- | --- | --- | --- | --- | --- | --- | --- | --- | --- | --- |
|  |  | **HR (95% CI)** | | **P-value** |  | **HR (95% CI)** | | **P-value** |  | **HR (95% CI)** | | **P-value** |
| Total cholesterol |  | 0.953 | (0.834-1.088) | 0.475 |  | 0.870 | (0.817-0.926) | <**0.001** |  | 0.875 | (0.790-0.970) | **0.011** |
| PRS |  | 1.014 | (0.895-1.149) | 0.831 |  | 1.039 | (0.828-1.304) | 0.739 |  | 1.040 | (0.955-1.133) | 0.369 |
| PRS * Total cholesterol |  | 1.034 | (0.928-1.151) | 0.548 |  | 1.004 | (0.823-1.224) | 0.971 |  | 1.026 | (0.951-1.107) | 0.502 |
| LDL-C |  | 0.957 | (0.836-1.095) | 0.518 |  | 0.867 | (0.814-0.923) | <**0.001** |  | 0.897 | (0.808-0.996) | **0.041** |
| PRS |  | 1.014 | (0.895-1.149) | 0.827 |  | 1.057 | (0.843-1.325) | 0.633 |  | 1.033 | (0.948-1.125) | 0.458 |
| PRS * LDL-C |  | 1.047 | (0.939-1.168) | 0.407 |  | 1.034 | (0.844-1.266) | 0.747 |  | 1.012 | (0.936-1.094) | 0.764 |
| HDL-C |  | 0.903 | (0.767-1.064) | 0.222 |  | 0.909 | (0.846-0.977) | **0.009** |  | 0.834 | (0.736-0.945) | **0.004** |
| PRS |  | 1.011 | (0.885-1.155) | 0.871 |  | 1.013 | (0.802-1.281) | 0.911 |  | 1.043 | (0.952-1.143) | 0.363 |
| PRS * HDL-C |  | 1.062 | (0.927-1.216) | 0.387 |  | 0.966 | (0.761-1.227) | 0.779 |  | 1.034 | (0.942-1.134) | 0.483 |
| Triglyceride |  | 1.136 | (1.010-1.277) | **0.034** |  | 1.039 | (0.987-1.093) | 0.141 |  | 1.038 | (0.944-1.141) | 0.444 |
| PRS |  | 0.997 | (0.884-1.125) | 0.963 |  | 1.051 | (0.846-1.307) | 0.652 |  | 1.019 | (0.936-1.109) | 0.668 |
| PRS * Triglyceride |  | 1.000 | (0.904-1.106) | 0.996 |  | 0.957 | (0.779-1.175) | 0.675 |  | 1.038 | (0.964-1.117) | 0.324 |

LDL-C, Low density lipoprotein cholesterol; HDL-C, High density lipoprotein cholesterol; PRS, polygenic risk score; HR, Hazard ratio; CI, confidence interval; SE, standard error.

Hazard ratios were reported as per 1-SD change for lipid levels.

Models including each lipid level, PRS as continuous variable, and multiplicative interaction term between them. Adjusted for age, sex, BMI, alcohol intake frequency, smoking status, comorbidities (diabetes, hypertension), statin use, fibrate use, fasting time, baseline eGFR, and top 10 genetic principal components

**Fig 2.** **Forest plot visualizing differential impacts of lipids on incident CKD according to genetic risk.** Multivariable analyses with model including each lipid as binary variable and PRS as tertiles were performed. Each model was adjusted for age, sex, BMI, alcohol intake frequency, smoking status, diabetes, hypertension, statin use, fibrate use, fasting time, baseline eGFR, the top 10 genetic principal components.

Participants who used fibrates totaled 731, accounting for 0.20% of the entire participants (Table 1). In the multivariable analysis utilizing Cox regression, the impact of triglycerides on the incident CKD remained consistent, with an HR of 1.082 (95% CI 1.052-1.113; p-value <0.001), showing no significant deviation from the original results (Table 2). Furthermore, there were no substantial changes in the influence of other lipid levels and PRS on incident CKD, as well as the trends in the interaction between PRS and lipid levels (Table 2, 3 and 4).
