# Supplementary material for: Association between dyslipidemia and the risk of incident chronic kidney disease affected by genetic susceptibility: Polygenic risk score analysis
Source: PLoS One. 2024 Apr 16;19(4):e0299605. doi: 10.1371/journal.pone.0299605 (PMC11020804; doi:10.1371/journal.pone.0299605)
Supplement: Attachment — Submitted filename: Rebuttal letter 0207ljh.docx [file pone.0299605.s008.docx]

**Comments from the Editor and Reviewers**

**Editor comments:**
Thank you for submitting your manuscript to PLOS ONE. After careful consideration, we feel that it has merit but does not fully meet PLOS ONE’s publication criteria as it currently stands. Therefore, we invite you to submit a revised version of the manuscript that addresses the points raised during the review process.

==============================

ACADEMIC EDITOR:

Previous comments have been addressed, but the manuscript will require minor revisions. While recognizing the potential interest of the subject studied, one of the reviewers raised additional comments that need to be properly addressed.

==============================

Please submit your revised manuscript by Mar 01 2024 11:59PM. If you will need more time than this to complete your revisions, please reply to this message or contact the journal office at plosone@plos.org. When you're ready to submit your revision, log on to https://www.editorialmanager.com/pone/ and select the 'Submissions Needing Revision' folder to locate your manuscript file.

Please include the following items when submitting your revised manuscript:

A rebuttal letter that responds to each point raised by the academic editor and reviewer(s). You should upload this letter as a separate file labeled 'Response to Reviewers'.

A marked-up copy of your manuscript that highlights changes made to the original version. You should upload this as a separate file labeled 'Revised Manuscript with Track Changes'.

An unmarked version of your revised paper without tracked changes. You should upload this as a separate file labeled 'Manuscript'.

If you would like to make changes to your financial disclosure, please include your updated statement in your cover letter. Guidelines for resubmitting your figure files are available below the reviewer comments at the end of this letter.

If applicable, we recommend that you deposit your laboratory protocols in protocols.io to enhance the reproducibility of your results. Protocols.io assigns your protocol its own identifier (DOI) so that it can be cited independently in the future. For instructions see: <https://journals.plos.org/plosone/s/submission-guidelines#loc-laboratory-protocols>. Additionally, PLOS ONE offers an option for publishing peer-reviewed Lab Protocol articles, which describe protocols hosted on protocols.io. Read more information on sharing protocols at <https://plos.org/protocols?utm_medium=editorial-email&utm_source=authorletters&utm_campaign=protocols>.

Please review your reference list to ensure that it is complete and correct. If you have cited papers that have been retracted, please include the rationale for doing so in the manuscript text, or remove these references and replace them with relevant current references. Any changes to the reference list should be mentioned in the rebuttal letter that accompanies your revised manuscript. If you need to cite a retracted article, indicate the article’s retracted status in the References list and also include a citation and full reference for the retraction notice.

**Response to Editor:**

Thank you for the comment. We have reviewed the reference list and found a correction in reference number 31. This correction was due to an error in electronic tagging and did not change the content of the article. However, after review, we changed the reference to a more appropriate reference and made the following changes to the reference list of the manuscript:

- Original Reference 31: Ebrahim S, Sung J, Song YM, Ferrer RL, Lawlor DA, Davey Smith G. Serum cholesterol, haemorrhagic stroke, ischaemic stroke, and myocardial infarction: Korean national health system prospective cohort study. Bmj. 2006;333(7557):22. Epub 2006/06/08. http://doi.org/10.1136/bmj.38855.610324.80
- Revised Reference 31: Miller M. Dyslipidemia and cardiovascular risk: the importance of early prevention. QJM. 2009;102(9):657-67. Epub 2009/06/04. http://doi.org/10.1093/qjmed/hcp065

**Reviewers' comments:**

**Reviewer #2:**

Thank you.
The authors addressed all my concerns.

**Reviewer #3:**

Thanks for your work on an interesting topic. You should underline more the limitations of the UK Biobank as history of diabetes and history of diabetes aren't sufficient enough to reflect the level of BP and/or glycemic time-exposure which should play a role in the occurence of kidney disease. Therefore the discussion should provide a complete overview of all biases for the readers, underlying that further specific research is warranted.

**Response to Reviewer #3:**

Thank you for the valuable comment. In response to the comment, we further explained the limitations of not having a time exposure factor for diabetes and hypertension in the Discussion section, emphasizing the need for further research: “Furthermore, information regarding the timing of diagnosis of diabetes and hypertension was not available from the UK Biobank, variables regarding the duration of exposure to hyperglycemia and high blood pressure, which are important in the development of kidney disease, could not be applied to the analysis. Further cohort studies, including more precise and regular assessments of clinical and lifestyle data incorporating temporal information, are warranted.
